# Einflussfaktoren der Schnitt-Naht-Zeiten der Metallentfernung nach Nuss-Operation

**DOI:** 10.1007/s00104-023-01914-w

**Published:** 2023-06-23

**Authors:** Andreas C. Heydweiller, Tatjana T. König, S. Tolga Yavuz, Martin Schwind, Christina Oetzmann von Sochaczewski, Stephan Rohleder

**Affiliations:** 1 Sektion Kinderchirurgie der Klinik und Poliklinik für Allgemein‑, Viszeral‑, Thorax- und Gefäßchirurgie, Universitätsklinikum Bonn, Bonn, Deutschland; 2 Klinik und Poliklinik für Kinderchirurgie, Universitätsmedizin Mainz, Mainz, Deutschland; 3 Klinik für Allgemeine Pädiatrie, Universitätsklinik Bonn, Bonn, Deutschland; 4 Sektion Kinderchirurgie, Klinik und Poliklinik für Allgemein‑, Viszeral‑, Thorax- und Gefäßchirurgie, Universitätsklinikum Bonn, Venusberg-Campus 1, 53127 Bonn, Deutschland

**Keywords:** Trichterbrust, Implantatentferung, Operationszeiten, Lineare Regression, Kinderchirurgie, Pectus excavatum, Implat removal, Surgical time, Linear regression, Pediatric surgery

## Abstract

**Hintergrund:**

Die Metallentfernung nach abgeschlossener Trichterbrustkorrektur fällt als elektiver Eingriff nicht selten zuerst den Kapazitätsengpässen der Operationsabteilung zum Opfer. Aufgrund dessen ist eine möglichst exakte Planung der zu erwartenden Schnitt-Naht-Zeit wünschenswert.

**Ziel der Arbeit:**

Modellierung der Schnitt-Naht-Zeiten der Metallentfernung nach Nuss-Operation anhand der präspezifizierten unabhängigen Variablen Alter, Geschlecht, Anzahl der zu explantierenden Metallbügel sowie intraoperativ aufgetretener Komplikationen.

**Material und Methoden:**

Wir schlossen retrospektiv alle Metallentfernungen nach Trichterbrustkorrektur zwischen Januar 2009 und Dezember 2020 in die Untersuchung ein. Diese wurden mittels linearer Regression modelliert und mittels Bootstrap intern validiert. Explorativ wurden zusätzlich die Erfahrung der Operateure, die Anzahl der Stabilisatoren sowie der Körpermasseindex und eine etwaige Revisionsoperation untersucht.

**Ergebnisse:**

Wir schlossen 265 Patient:innen (14 % ♀) mit einem medianen Alter von 19 Jahren (Interquartilsabstand: 17–20) in die Untersuchung ein, wobei bei 81 % ein und bei 17 % zwei Metallbügel explantiert wurden. Das präspezifizierte Regressionsmodell war statistisch signifikant besser als das Nullmodell (Likelihood-Ratio 56; df = 5; *p* < 0,001) und hatte eine biaskorrigierte Modellgüte von *R*^2^ = 0,148. Das Patient:innenalter beeinflusste die Schnitt-Naht-Zeit um 2,1 min (95 %-Konfidenzintervall: 1,3–2,9; *p* < 0,001) pro Lebensjahr und jeder zu explantierende Metallbügel um 16 min (95 %-Konfidenzintervall: 10–22; *p* < 0,001).

**Schlussfolgerung:**

Das Patient:innenalter wie auch der Anzahl der zu explantierenden Metallbügel können die Schnitt-Naht-Zeit beeinflussen und können in der Zeitplanung der Operation Berücksichtigung finden.

## Abstract (en)

**Background:**

Metal bar removal after the Nuss repair procedure is prone to be cancelled in cases of operating time shortages due it being suitable to be postponed without harming patients. Consequently, planning operation times as exactly as possible could be one solution.

**Objective:**

Statistical modelling of operation times of metal bar removal after Nuss repair using the prespecified independent predictors of age, sex, intraoperative complications, and number of implanted metal bars.

**Material and methods:**

We included all patients whose operation notes included an operation time, which was modelled via linear regression and subject to internal validation via bootstrap. Exploratory analyses also consisted of the surgeon’s experience, the number of stabilizers, the body mass index, and preceding re-do surgery for bar dislocation.

**Results:**

We included 265 patients (14% ♀) with a median age of 19 years (interquartile range 17–20 years), of whom 81% had 1 and 17% had 2 metal bars removed. The prespecified regression model was statistically significant (likelihood ratio 56; df = 5; *P* < 0.001) and had a bias corrected *R*^2^ of 0.148. Patient age influenced operation times by 2.1min per year of life (95% confidence interval 1.3–2.9min; *P* < 0.001) and 16min per explanted metal bar (95% confidence interval: 10–22min; *P* < 0.001).

**Conclusion:**

The patient-specific factors of age and the number of explanted metal bars influenced the operation times and can be included into scheduling operation times.

## Hintergrund und Fragestellung

Verschiedentlich wurde, auch in dieser Zeitschrift [1], beklagt, dass bestimmte Eingriffe nicht mehr kostendeckend durchzuführen wären, was insbesondere Universitätskliniken beträfe [2, 3]. Eine Schlüsselrolle haben dabei die Schnitt-Naht-Zeiten. Diese sind, neben den anderen Prozesszeiten, untrennbar verbunden mit der Auslastung der Betriebszeiten der Operationsabteilung als einer der wesentlichen Erlösbringer eines Krankenhauses im DRG-System [4]. Die Operationsplanung zielt daher darauf ab, die zur Verfügung stehenden Betriebszeiten möglichst effizient auszulasten. In der Planung ist dabei neben einer Reduktion der Überleitungszeiten eine präzise Vorhersage der zu erwartenden Operationsdauer von Relevanz [5]. Zu niedrig geplante Schnitt-Naht-Zeiten, die ständig überschritten werden, sind der Zufriedenheit der Mitarbeiter:innen wie auch der Patient:innen abträglich [6]. Zwar bilden sich Erfahrungswerte für die durchschnittliche Dauer bestimmter Operationen heraus, jedoch ist die Vorhersagequalität oftmals schwach, insbesondere hinsichtlich eines zu geringen Zeitansatzes, was sich durch alle Fachgebiete zieht [7]. Es sind gerade elektive Eingriffe, die sich für eine Analyse möglicher Einflussfaktoren der Schnitt-Naht-Zeit eignen, da sowohl Spezifika der Operation selbst wie auch patientenbezogene Faktoren die Schnitt-Naht-Zeit beeinflussen [8]. Zu diesen elektiven Eingriffen zählt auch die Metallentfernung nach abgeschlossener Trichterbrustkorrektur im Verfahren nach Nuss [9]. Die Operationszeit selbst wurde nur gelegentlich betrachtet, wobei an einem kleinen Kollektiv beschrieben wurde, dass sich die Operationsdauer umgekehrt proportional zum Ausbildungsstand verlängert [10]. Dies ist jedoch keine Besonderheit der Metallentfernung nach abgeschlossener Trichterbrustkorrektur, sondern für eine Vielzahl an Operationen beschrieben [11]. Wir untersuchten daher systematisch die präspezifizierten Einflussfaktoren der Schnitt-Naht-Zeit der Metallentfernung nach abgeschlossener Trichterbrustkorrektur.

## Studiendesign und Untersuchungsmethoden

Wir identifizierten retrospektiv alle Metallentfernungen nach abgeschlossener Trichterbrustkorrektur im Verfahren nach Nuss über die OPS-Kodierung 5‑349.5 sowie, für das Jahr 2009 vor Einführung dieses Codes, alle Patienten mit dem Diagnosecode für die Trichterbrust (ICD-10-GM Q67.0) durch Prüfung aller Operationsberichte, im Zeitraum zwischen dem 01.01.2009 und dem 31.12.2020 in beiden beteiligten kinderchirurgischen Kliniken aus Bonn und Mainz. Patient:innen wurden eingeschlossen, sofern eine gültige Schnitt-Naht-Zeit dokumentiert war. Von den eingeschlossenen Fällen wurden das Alter zum Operationszeitpunkt, das Geschlecht, die Anzahl der entfernten Metallbügel, etwaige intraoperative Komplikationen und schließlich die Krankenhausverweildauer erfasst. Die Datenerfassung erfolgte nach gesonderter Einweisung anhand eines präspezifizierten Datenblatts, dessen Tauglichkeit in einer Zufallsstichprobe geprüft war. Überdies wurden stichprobenartig die Ergebnisse der Datenerfassung durch einen anderen beteiligten Autor überprüft, um eine konsistente Datenqualität sicherzustellen. In der Jurisdiktion unserer Ethikkommissionen besteht bei rein retrospektiver Datenerfassung von Routinedaten mit Anonymisierung an der Quelle, hier dem Krankenhausinformationssystem, kein Beratungsbedarf.

Alle statistischen Analysen dieses Projekts waren präspezifiziert, soweit nicht anders berichtet. Für die Durchführung der statistischen Berechnungen verwendeten wir R (Version 3.5.3) mit dem stats4-Paket, soweit nicht anders angegeben [12]. Mediane wurden mittels des Westenberg-Mood-Mediantestes verglichen und Unterschiede zwischen den Geschlechtern hinsichtlich der Anzahl der implantierten Metallbügel mittels χ^2^-Test evaluiert. Der Einfluss der einbezogenen Faktoren Patientenalter und -geschlecht, Anzahl der entfernten Metallbügel und Auftreten intraoperativer Komplikationen auf die Schnitt-Naht-Zeit wurde mittels multivariabler linearer Regression der kleinsten Quadrate evaluiert, wofür wir das rms-package (Version 6.3‑0; [13]) verwendeten. Aufgrund der kürzlich berichteten Tatsache, dass sich Patientinnen im Durchschnitt im höheren Lebensalter sowie mit einem höheren Schweregrad der Erkrankung vorstellten [14], ergänzten wir die Regressionsanalyse um den Interaktionseffekt von Alter und Geschlecht. Die Normalverteilung der Residuen wurde mittels visueller Analyse des Quantil-Quantil-Diagramms sowie der Cook-Distanz überprüft. Um eine Reproduzierbarkeit des Bootstraps zu gewährleisten, legten wir den Startpunkt des Zufallsgenerators auf 1337 fest. Aufgrund der präspezifizierten unabhängigen Variablen nutzten wir die interne Validierung mittels Bootstrap mit 1000 Iterationen, um für das Bias der Modellgüte zu korrigieren [15], wofür wir ebenfalls das rms-package verwendeten. Dementsprechend legten wir für die 95 %-Konfidenzintervalle ebenfalls die Bootstrapschätzer zugrunde, allerdings berechnet mit 10.000 Iterationen. Da für die unabhängige Variable des Patient:innenalters kein Wert von 0 denkbar ist, zentrierten wir das Regressionsmodell mittels der center_mod-Funktion des jtools-packages (Version 2.0.2; [16]).

Im Rahmen des Begutachtungsverfahrens der Arbeit wurden zusätzliche explorative Analysen zur Berücksichtigung des Einflusses des Ausbildungsstandes der Operateure, einer etwaigen vorangegangenen Revisionsoperation sowie zur Anzahl der entfernten Stabilisatoren und des Körpermasseindexes der Patient:innen eingeführt. Die Analyse erfolgte dabei nach dem bereits beschriebenen Vorgehen.

## Ergebnisse

Wir schlossen 265 durchgeführte Metallentfernungen mit gültiger Schnitt-Naht-Zeit in unsere Analyse ein, davon wurden 227 (86 %) bei Patienten und 38 (14 %) bei Patientinnen durchgeführt. Das mediane Alter der eingeschlossenen Patient:innen betrug 19 Jahre (Interquartilsabstand: 17–20), wobei Patientinnen mit im Median 18 Jahren (Interquartilsabstand: 16–20) etwas jünger waren als Patienten (z = −2,9; *p* = 0,004), deren medianes Alter 19 Jahre (Interquartilsabstand: 18–20) betrug. Bei 215 Patient:innen (17 % ♀) war ein Metallbügel implantiert, bei 46 (15 % ♀) waren es derer zwei und bei 4 Patienten drei Metallbügel, wobei zwischen den Geschlechtern kein Unterschied bestand (χ^2^ = 0,7; df = 2; *p* = 0,705). Intraoperative Komplikationen jeglicher Art, je zweimal ausgeprägte Ossifikationen und zwei Pneumothoraces sowie zweimalige kleine Drahtreste, traten bei 6 (2 %) Patient:innen (5 ♂; 1 ♀) auf. Die mediane Krankenhausverweildauer unserer Patientenkohorte betrug 2 Tage (Interquartilsabstand: 1–2). Revisionsoperationen waren bei 12 Patienten (5 %) und 6 Patientinnen (16 %) erforderlich gewesen. Die meisten Metallentfernungen wurden durch Oberärzt:innen (*n* = 136, 51 %) und Chefärzte (*n* = 117, 44 %) durchgeführt, wohingegen Fachärzt:innen (*n* = 5, 2 %) und Weiterbildungsassistent:innen (*n* = 7, 3 %) als Durchführende die Ausnahme waren. Es wurden mehrheitlich zwei Stabilisatoren (*n* = 194, 73 %) und seltener ein Stabilisator (*n* = 60, 23 %) verwendet, wohingegen die Verwendung keines Stabilisators (*n* = 6, 2 %) sowie von drei (*n* = 3, 2 %) oder vier (*n* = 2, 1 %) Stabilisatoren die Ausnahme blieb. Informationen hinsichtlich des Körpermasseindexes waren zu 221 Patient:innen (83 %) verfügbar, welcher im Median 20,2 kg/m^2^ (Interquartilsabstand: 18,4–21,7) betrug.

Die Regressionsanalyse unter Einbezug der präspezifizierten unabhängigen Variablen erwies sich als treffsicherer als das Nullmodell, welches keine der eingeschlossenen unabhängigen Variablen beinhaltete, sondern nur den Achsenabschnitt, bei dem alle Regressionsparameter den Wert Null annehmen, (Likelihood-Ratio 56; df = 5; *p* < 0,001) und das Bestimmtheitsmaß *R*^2^ betrug 0,192. Die interne Validierung mittels Bootstrap zeigte ein Bias der Modellgüte von 0,044, weshalb das korrigierte *R*^2^ den Wert von 0,148 annahm. Im Vergleich der Modelle erwies sich das Modell mit den explorativ geprüften unabhängigen Variablen als geringfügig besser (*F*[254,5] = 3,692; *p* = 0,003). Das Bias der Modellgüte betrug 0,079, weshalb sich das *R*^2^ von 0,247 auf den korrigierten Wert von 0,168 durch interne Validierung reduzierte. Die Hinzunahme der unabhängigen Variable des Körpermasseindexes erhöhte das mittels interner Validierung korrigierte *R*^2^ nur minimal auf 0,171.

**Figure 1:**
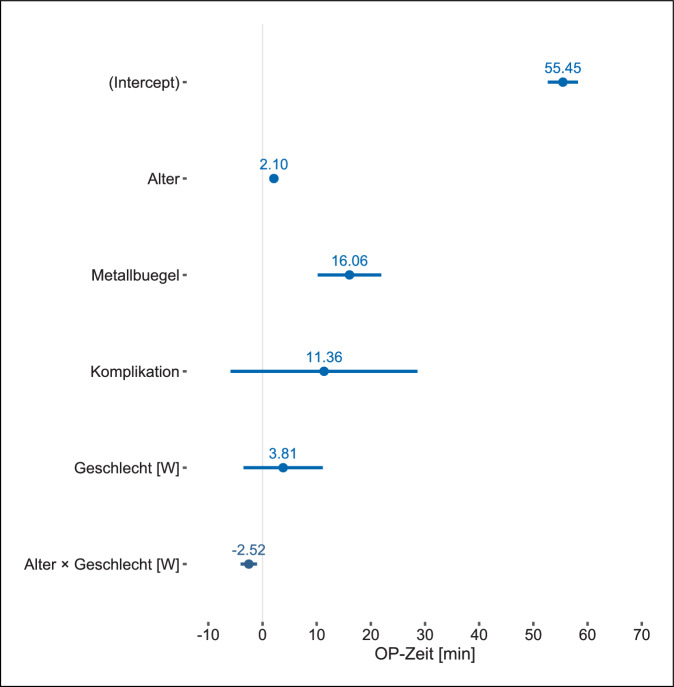

**Figure 2:**
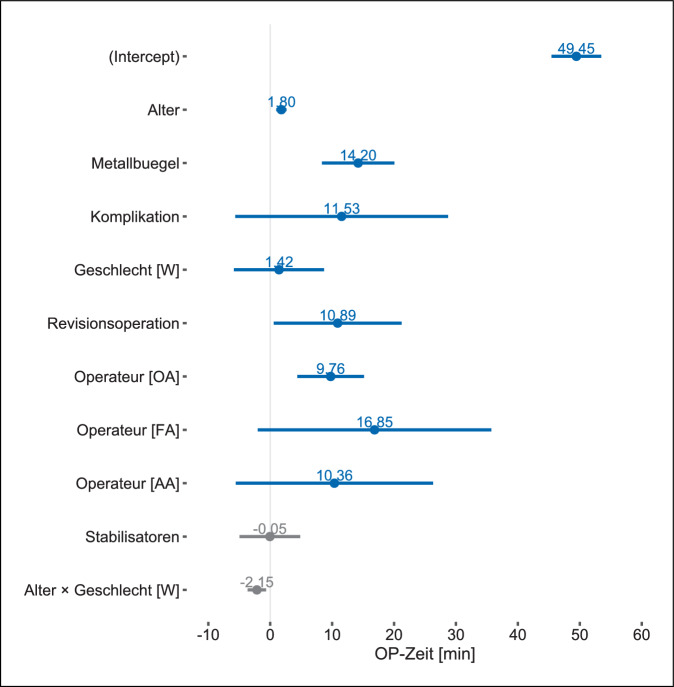

**Figure 3:**
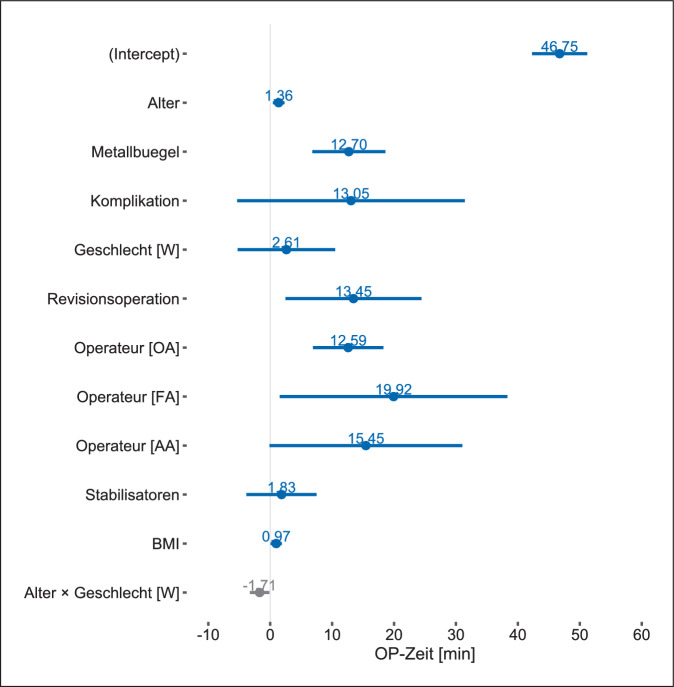

Sowohl im präspezifizierten Modell (Abb. 1) wie auch in den explorativen Modellen (Abb. 2 und 3) erwies sich das Patient:innenalter und die Anzahl der Metallbügel als relevanter Prädiktor für die Schnitt-Naht-Zeit. In den explorativen Modellen war lediglich die Durchführung der Metallentfernung durch Oberärzt:innen, im Vergleich zur Referenzkategorie der Chefärzte, mit einer längeren Schnitt-Naht-Zeit assoziiert (Abb. 2 und 3). Dies traf für die Metallentfernung durch Fach:ärztinnen und Weiterbildungsassistent:innen nicht zu, allerdings dürfte die Aussagekraft durch die geringe Fallzahl beschränkt sein. Der Effekt des Körpermassenindex war nachzuweisen, jedoch im Ausmaß überschaubar (Abb. 3), im Gegensatz zur Revisionsoperation, die, sofern in der Vorgeschichte dokumentiert, einen deutlichen Einfluss auf die Schnitt-Naht-Zeit ausübte (Abb. 2 und 3). Hinsichtlich der Anzahl der Stabilisatoren ließ sich kein Effekt zeigen (Abb. 2 und 3).

**Figure 4:**
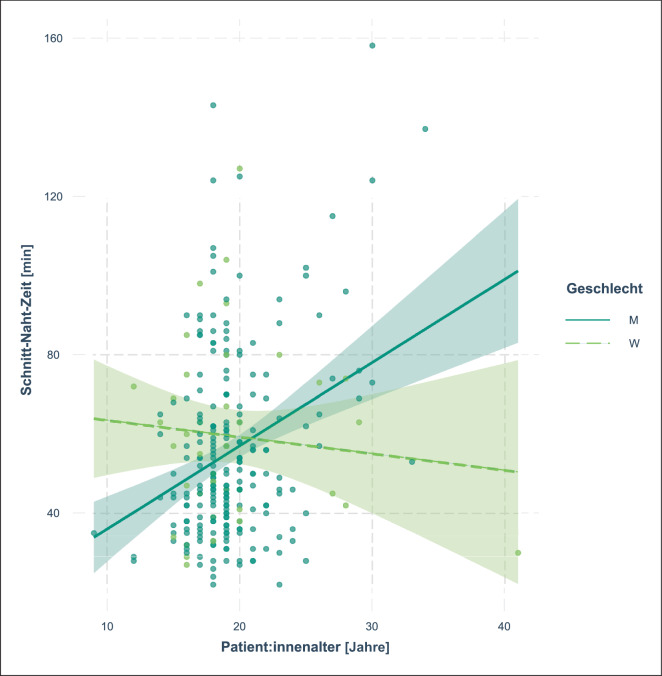

Die Relevanz des Interaktionseffekts, welcher sich ebenfalls in allen Modellen nachweisen ließ, lässt sich besser grafisch darstellen (Abb. 4) und demonstriert die Abnahme der Schnitt-Naht-Zeit bei Patientinnen mit zunehmendem Alter im Gegensatz zum Kurvenverlauf für Patienten.

## Diskussion

Nicht wenige Eingriffstypen sind, insbesondere im universitären Bereich, noch kostendeckend durchzuführen [1–3] und als planbare Operationen sowohl von äußeren Einflüssen wie der, bislang singulären, COVID-19-Pandemie, aber auch von Streikmaßnahmen und insbesondere Personalmangel besonders betroffen [17]. Neben prozessorganisatorischen Verbesserungen wird häufig die Reduktion der Schnitt-Naht-Zeit als relevante Komponente angesehen [3, 5, 18]. Zu den elektiven Eingriffen gehört auch die Metallentfernung nach abgeschlossener Trichterbrustkorrektur nach der Nuss-Methode [9]. Bisher war lediglich beschrieben worden, dass die Schnitt-Naht-Zeit der Metallentfernung sich umgekehrt proportional zum Ausbildungsstand der Operierenden verhielt [10].

Wir wählten die untersuchten präspezifizierten, unabhängigen Variablen anhand der Literatur zum untersuchten Eingriff aus: Media und Mitarbeiter:innen beschrieben, dass auftretende Komplikationen die Schnitt-Naht-Zeit der Metallentfernung nach abgeschlossener Trichterbrustkorrektur im Verfahren nach Nuss verlängerten [19]. So wenig überraschend dieses Ergebnis auch ist, so beschrieben die Autoren auch, dass Patient:innen, die eine Komplikation erlitten, im Schnitt älter waren und überdies ein Zusammenhang zwischen Patient:innenalter und dem Auftreten intraoperativer Komplikationen bestand [19], was auch in anderen Arbeiten beschrieben war [20]. Zwar wurde in dieser Untersuchung nicht untersucht, ob das Alter nur über den Faktor der Komplikationshäufigkeit die Schnitt-Naht-Zeit verlängert, sondern möglicherweise auch ein kovariater Faktor der Schnitt-Naht-Zeit sein könnte. Dies ist zumindest naheliegend, da ältere Patient:innen einen deutlich steiferen Thorax aufweisen [21], was ein Faktor einer verlängerten Schnitt-Naht-Zeit sein könnte. Unsere Daten scheinen diese Annahme zu bestätigen, da sie einen Zusammenhang zwischen dem Patient:innenalter und der Schnitt-Naht-Zeit zeigen.

Aufgrund des beschriebenen Einflusses intraoperativer Komplikationen auf die Schnitt-Naht-Zeit berücksichtigten wir diese ebenfalls [19, 20]. Dieses Ergebnis ließ sich anhand unserer Daten nicht bestätigen, allerdings war sowohl die relative als auch die absolute Häufigkeit intraoperativer Komplikationen in diesen Untersuchungen deutlich höher als in unserer Kohorte [22]. Daher könnte die Effektstärke in der linearen Regression möglicherweise nicht mehr hoch genug sein, allerdings konnten Simulationsstudien zeigen, dass bei der multivariablen linearen Regression mittels der Methode der kleinsten Quadrate bereits zwei Ereignisse ausreichend sind, um verlässliche Punktschätzer und Konfidenzintervalle zu berechnen [23].

Die Anzahl der zu explantierenden Metallbügel war in der Untersuchung von Nyboe und Kolleg:innen neben dem bereits genannten Ausbildungsstand sowie der Kallusbildung am Implantat ein wesentlicher Einflussfaktor der Schnitt-Naht-Zeit [10]. Zwar ist die Datenlage in der Literatur hierzu nicht eindeutig, da andere Arbeiten zeigten, dass die Anzahl der implantierten Metallbügel keinen Einfluss auf die Schnitt-Naht-Zeiten hatte [24], jedoch war in dieser Untersuchung die Variable dichotomisiert worden, was mit einer deutlichen Abnahme der Trennschärfe der Untersuchung einhergeht [25]. Dies mag mit ein Faktor sein, dass sich in unserer Untersuchung wiederum ein relativ deutlicher Effekt der Anzahl der explantierten Metallbügel nachweisen ließ, da wir die abzählbare Variable auch als kontinuierliche Variable in der Regressionsanalyse behandelten. Im Gegensatz zur Anzahl der explantierten Metallbügel erwies sich die Anzahl der entfernten Stabilisatoren als irrelevant für die Schnitt-Naht-Zeit in unserer explorativen Analyse. Dies stimmt mit den bisherigen Beschreibungen aus der Literatur überein, die einen Einfluss auf die Komplexität der Metallentfernung nur aus den Anfangstagen der Nuss-Operation beschrieben [26].

Wir berücksichtigten überdies das Geschlecht der Patient:innen, da dies bislang, aufgrund der geringeren Prävalenz der Erkrankung bei Frauen und Mädchen, ein wenig berücksichtigter Aspekt ist und neuere Untersuchungen zeigten, dass sich Patientinnen in höherem Alter und mit stärker ausgeprägter Symptomatik zur operativen Korrektur vorstellten [14]. Aufgrund dessen gingen wir von einer möglichen Verzerrung durch diesen Effekt aus und berücksichtigten ihn mittels des Interaktionsterms von Geschlecht und Alter und konnten auch eine Interaktion nachweisen. Jedoch erachten wir diese Interaktion trotz der statistischen Signifikanz für wenig verlässlich, da in unserer Kohorte 16 % der Patientinnen über 25 Jahre alt waren, jedoch nur 6 % der Patienten, mithin die lebensälteren Patienten unterrepräsentiert sind. Allerdings ist die absolute Anzahl der Patienten höher ist als die der Patientinnen, was sich im insgesamt schmaleren Konfidenzintervall widerspiegelt, auch wenn dieses gerade im Bereich des höheren Lebensalters deutlich breiter wird. Dies mag ein Effekt davon sein, dass an unseren Kliniken die Trichterbrustkorrektur aufgrund der historischen Zuordnung zur Kinderchirurgie, infolge der früher bei der offenen Korrektur im Regelfall deutlich jüngeren Patient:innen, auch bei Erwachsenen in kinderchirurgischer Hand verblieben ist. Infolgedessen scheint eine Übertragbarkeit auf Kliniken, die vornehmlich Erwachsene, gerade unter Anwendung der für diese nicht unüblichen technischen Modifikationen der Operation [9], behandeln, zumindest fraglich.

Hinsichtlich der explorativ untersuchten Faktoren zeigte sich der erwartete Effekt [11]: Bezogen auf die Referenzkategorie der Chefärzte verlängerte sich die Schnitt-Naht-Zeit, wenn Oberärzt:innen operierten. Numerisch war dies auch bei Fachärzt:innen und Weiterbildungsassistent:innen der Fall, allerdings sind diese Ergebnisse aufgrund der geringen Anzahl in unserer Kohorte mit einer relevanten Unsicherheit behaftet. Der Zusammenhang zwischen dem Ausbildungsstand der Operateur:innen und der Schnitt-Naht-Zeit war von Nyboe und Mitarbeiter:innen bereits berichtet worden, wenngleich bezogen auf den Vergleich zwischen Weiterbildungsassistent:innen unterschiedlicher Erfahrungsstufen [10]. Unsere Ergebnisse deuten mithin darauf hin, dass es auch bei der eingriffsspezifischen Erfahrung eine anhaltende Lernkurve zu geben scheint.

Wir sahen in unserer Kohorte einen statistisch signifikanten Effekt der Revisionsoperation auf die Schnitt-Naht-Zeit. Zwar wurde dieser noch nicht direkt nachgewiesen, allerdings wurde ein Zusammenhang zwischen Bügeldislokationen und dem Auftreten mehr als unerheblicher Komplikationen gezeigt [27]. Diese sind mit komplizierteren und technisch anspruchsvollen Metallentfernungen vergesellschaftet [28], sodass sich daraus längere Schnitt-Naht-Zeiten bei der Metallentfernung ergeben könnten.

Weitere Einschränkungen der Übertragbarkeit unserer Ergebnisse ergeben sich zwangsläufig aus dem retrospektiven Studiendesign wie auch aus der fehlenden Berücksichtigung möglicher relevanter Kovariate infolge der präspezifizierten unabhängigen Variablen. So ist beispielsweise beschrieben, dass ein Körpermasseindex jenseits der 22 kg/m^2^ zu einer Verlängerung der Schnitt-Naht-Zeit führt [24], allerdings leidet auch dieses Ergebnis am Problem der Dichotomisierung einer kontinuierlichen Variable [25]. Zwar fehlten in unserer Kohorte bei 17 % die Informationen, um den Körpermasseindex zu berechnen, dennoch konnten auch wir einen Effekt des Körpermasseindexes auf die Schnitt-Naht-Zeit nachweisen. Erwartungsgemäß war dieser, aufgrund der geringen Spannweite des Körpermasseindex bei unseren Patent:innen, hinsichtlich der Effektstärke überschaubar. Auch für andere Eingriffstypen, beispielsweise die proximale Femurfraktur, konnte gezeigt werden, dass ein steigender Körpermasseindex sich proportional zur ansteigenden Schnitt-Naht-Zeit verhält [29].

Die Dauer der Bügeleinlage wurde nicht für die Vorhersage berücksichtigt, da diese sowohl in älteren [10, 24] wie auch in neueren [30] Arbeiten keinen Einfluss auf die Operationszeit der Metallentfernung hatte. Für eine explorative automatisierte Selektion möglicher Prädiktoren, beispielsweise mittels Vorwärtsselektion, erschien uns unsere Kohorte zu klein, sodass aufgrund des zu erwartenden Mangels an statistischer Power kaum verlässliche Ergebnisse hätten erzielt werden können. Infolgedessen fokussierten wir unsere Untersuchung auf die Faktoren, die bereits einen Anknüpfungspunkt in der bisher veröffentlichten Literatur hatten, und präspezifizierten unsere unabhängigen Variablen anhand des bisherigen Wissensstandes in der Literatur.

## Fazit für die Praxis

- Die Schnitt-Naht-Zeiten der Metallentfernung nach abgeschlossener Trichterbrustkorrektur nach der Nuss-Methode lässt sich statistisch modellieren.
- Die Berücksichtigung der patienteneigenen Faktoren von Alter und Anzahl der zu explantierenden Metallbügel kann bei der Planung der Schnitt-Naht-Zeit berücksichtigt werden.
- Dies gilt möglicherweise ebenfalls für die vorangegangene Revisionsoperation sowie vermutlich für die individuelle eingriffsspezifische Erfahrung der Operateure und den Körpermasseindex, jedoch sind für sichere Aussagen hierzu noch bestätigende Studien erforderlich.
